# Supplementary figures and images for: Proof-of-concept for a non-invasive, portable, and wireless device for cardiovascular monitoring in pediatric patients
Source: PLoS One. 2020 Jan 3;15(1):e0227145. doi: 10.1371/journal.pone.0227145 (PMC6941801; doi:10.1371/journal.pone.0227145)

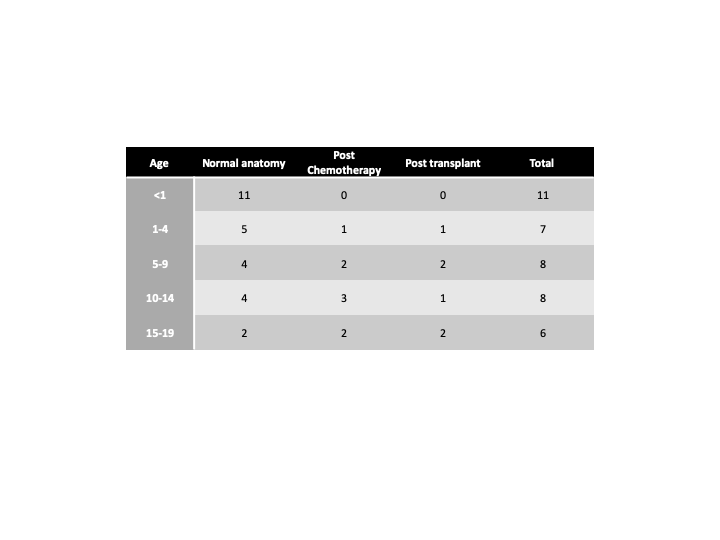

Supplement: S1 Table — Number of patients with each type of physiology by age group. (TIFF) [file pone.0227145.s001.tiff]
