# Supplementary material for: Proof-of-concept for a non-invasive, portable, and wireless device for cardiovascular monitoring in pediatric patients
Source: PLoS One. 2020 Jan 3;15(1):e0227145. doi: 10.1371/journal.pone.0227145 (PMC6941801; doi:10.1371/journal.pone.0227145)
Supplement: Attachment — Submitted filename: Response to Reviewers.docx [file pone.0227145.s002.docx]

**AUTHORS’ RESPONSES TO REVIEWERS’ SUGGESTIONS**

**Manuscript #:** PONE-D-19-22751

**Title:** Proof-Of-Concept For A Non-invasive, Portable, and Wireless Device for Cardiovascular Monitoring in Pediatric Patients

**Authors:** Jennifer C. Miller, Jennifer Shepherd, Derek Rinderknecht, Andrew Cheng, Niema M. Pahlevan

We thank our editor and reviewers for the positive evaluations and constructive suggestions. Our manuscript has been greatly improved with the reviewers’ help. Below we list our responses in the sequence in which they were raised in each reviewer’s report.

**Journal Requirements:**

**Journal requirement 1.** When submitting your revision, we need you to address these additional requirements

Please ensure that your manuscript meets PLOS ONE's style requirements, including those for file naming. The PLOS ONE style templates can be found at *http://www.journals.plos.org/plosone/s/file?id=wjVg/PLOSOne_formatting_sample_main_body.pdf and http://www.journals.plos.org/plosone/s/file?id=ba62/PLOSOne_formatting_sample_title_authors_affiliations.pdf*

**Response:**

Please see edits to the draft per above guidelines. Specifically, the names of authors have been changed to first name followed by last name with “,” instead of “;”. The zip code has been removed from the institution department names. Colons have been removed following large titles.

**Journal requirement 2.**  Thank you for including your ethics statement:  "This study was approved by the CHLA IRB CHLA-17-00377. Consent/assent was obtained verbally form the participant and/or their parent as appropriate.".

a.Please amend your current ethics statement to include the full name of the ethics committee/institutional review board(s) that approved your specific study.

b.Once you have amended this/these statement(s) in the Methods section of the manuscript, please add the same text to the “Ethics Statement” field of the submission form (via “Edit Submission

**Response:**

The edits have been made as requested.

**Journal requirement 3.**  Thank you for stating in your Funding Statement:

"N.P.: This study was partially funded by Atlantic Pediatric Device Consortium (APDC) Subaward No. RG219-G6 <http://atlanticpediatricdeviceconsortium.org/> The funders had no role in the study design, data collection and analysis, decision to publish, or preparation of the manuscript."

Please provide an amended statement that declares *all* the funding or sources of support (whether external or internal to your organization) received during this study, as detailed online in our guide for authors at http://journals.plos.org/plosone/s/submit-now.  Please also include the statement “There was no additional external funding received for this study.” in your updated Funding Statement. Please include your amended Funding Statement within your cover letter. We will change the online submission form on your behalf.

**Response:**

The following updated statement has been added to the cover letter.

“This study was partially funded by Atlantic Pediatric Device Consortium (APDC) Subaward No. RG219-G6 <http://atlanticpediatricdeviceconsortium.org/>. N.P. received this award. The funders had no role in the study design, data collection and analysis, decision to publish, or preparation of the manuscript. There was no additional external funding received for this study.”

**Journal requirement 4**: Thank you for stating the following in the Competing Interests section:

"Niema M. Pahlevan hold equity in Avicena LLC  and has consulting agreement with Avicena LLC.

Derek Rinderknecht is the Chief Technical Officer of Avicena, LLC, the manufacturer of the Vivio and owns equity stake in the company."

Please confirm that this does not alter your adherence to all PLOS ONE policies on sharing data and materials, by including the following statement: "This does not alter our adherence to  PLOS ONE policies on sharing data and materials.” (as detailed online in our guide for authors http://journals.plos.org/plosone/s/competing-interests).  If there are restrictions on sharing of data and/or materials, please state these. Please note that we cannot proceed with consideration of your article until this information has been declared.

Please include your updated Competing Interests statement in your cover letter; we will change the online submission form on your behalf.

**Response:**

The following updated statement has been added to the cover letter.

“Niema M. Pahlevan holds equity in Avicena LLC and has a consulting agreement with Avicena LLC. Derek Rinderknecht is the Chief Technical Officer of Avicena, LLC, the manufacturer of the Vivio device, and owns equity stake in the company. This does not alter our adherence to PLOS ONE policies on sharing data and materials.”

**Comments to the Author**
***Reviewer 1:***

**Comment 1**: A further discussion of the challenges with the device in younger children would be helpful to the reader.

**Response:**

Thank you for your review and the thoughtful comments. We have revised the Discussion in the manuscript as follows to further discuss the challenges of the device in younger children.

“The Vivio successfully captured the carotid artery waveform in all children greater than 5 years of age. The Vivio best captured the carotid artery waveform when the subject held his/her neck in a position to expose the carotid triangle. This was achieved by rotating the head laterally 30-60 degrees and tilting the head up about 30 degrees. Neonates have a characteristically shorter neck in part secondary to a larger head to body ratio and lack of muscle development, and thus require a more significant superior tilt of the chin up to 60 degrees to expose the carotid triangle. The Vivio was successful in capturing the carotid artery waveform in children less than 5 years who were able to hold still with their necks in this position without making noise, as motion and sound create significant artifact. Methods to achieve this position included placing a neck roll beneath the back in the case of neonates, positioning the child in the parents lap sideways and leaning the head on the parent’s shoulder, and having the child sit in the parent’s lap with a video of interest positioned slightly up and to the side to achieve the angle of the neck desired. The device itself was less irritating to younger children than the process of finding the carotid pulse, and so it is possible the success rate may have significantly improved if the operator spent more time (e.g. 5 min) measuring for the waveform after giving the child time to settle. Furthermore, younger children are often fearful of strangers as well as unknown devices, particularly in unfamiliar settings, leading to significant motion artifact. It is likely that with parental training this device can be used by parents with whom the patients are more comfortable or in the setting of sleep at home. Parents were amenable to using the device themselves based on the simple application of the device.”

***Reviewer 2:***

**Comment 1a:** METHODS: For the age groupings do the authors actually mean 1-4, 5-9, 10-15, 16-20? There seemed to be overlap in the age groups 1-5, 5-10, 10-15, 15-20 in some places and >15 in others, 16-20.

**Response:**

We would like to thank the reviewer for this constructive comment. We have changed the group labels to <1, 1-4, 5-9, 10-14, and 15-19. These adjustments were made throughout in the document, tables, and figures as necessary. For further clarification, in the Methods we stated that we enrolled patients 0-19.9 years old and additionally defined age groups as follows so there would be no confusion about age cut offs:

“The three Vivio heads were compared in the age groups <1, 1-4 (1.0-4.99), 5-9 (5.0-9.99) and 10-14 (10.0-14.99) years of age to see if there was an advantage of using one head size over the other based on age.” . . . . “Children 15 – 19 (15.0-19.99) years old were presumed to be adult-sized and not included in this comparison. The standard head size was used for these patients”

In the Results section we included:

“Forty patients were enrolled with a median age of 6.7 years with a range of 0-19.4 years.”

**Comment 1b**: However, both probes appear to have been used in the 15 and under, but only the standard adult probe in the 16 and older group. Please clarify, otherwise it appears that some ages are double counted and “groupings” seem off.

**Response:**

In the original analysis, we included one patient age 15.4 years old in the comparison of the 3 head sizes. For this reason, we stated we only used the standard probe in patients 16 and older. Removing this patient does not alter our overall conclusion that there was successful capture of analyzable waveform in all patients age 10-14. We thus removed this patient and recreated the Fig 3 in order to keep consistent age cohorts throughout the paper to minimize confusion. Removal of the 15.4 year-old patient resulted in all patients with computer analyzable signals using both medium and large heads. This adjustment was made to Fig 3a. There were no changes to Fig 3b.

In making this edit, we noted that Fig 3 was created based on the original and not final computer averaged data set. The final computer averaged data set used for analysis in the rest of the paper was able to measure a waveform using both the standard and large head in a patient previously only noted have a wave form measured in the large head in the age group 5-9 years. This adjustment was made in Fig 3a. There was no difference in Fig 3b or to the conclusion described in the paper that only 80% of patients had signals that were analyzable by computer algorithm alone.

**Comment 2:** RESULTS: Despite finding statistically significant differences between children and adults the numbers in each pediatric group are quite small, especially in the less than 5 age group, in which only 3-5 patients had an analyzable wave forms. I am not sure the frequency data (IF) from the Vivio should even be reported for the less than 5 age group, with larger numbers statistical significance could actually be lost.

**Response:**

We would like to thank the reviewer for this constructive comment. When the less than 5 years of age group is removed, there is a significant difference in ω_1_ between the pediatric and adults cohorts (prob>F 0.0027). Post-hoc analysis demonstrates the difference is between the adult and age 15-19 cohort (p=0.003). Thus, removal of the age cohort 0-4 does not change the conclusion in regards to ω_1_ and we will need to further data in the future to deduce an equation for LVEF. Removal of the age group less than 5 years of age shows that there is not a significant difference in ω_2_ between the adult and pediatric cohorts (prob>F 0.16). This is in line with the subgroup analysis provided in the paper that demonstrates a significant difference only between the age cohorts 0-4 and the adult population. We agree that keeping this population in our analysis has the possibility to alter the results of ω_2_; however, given that there is still a difference in ω_1_ and the fact that this population in particular is in need of a better method for measuring LVEF, we feel it is important to include the age cohort in our analysis. We have modified our discussion to address the reviewers concern as follows:

“It is important to note that removal of the cohort age 0-4 may change the statistical significance of ω_1_ and ω_2_ particularly with larger cohort sizes; however, given the great need for a better method to measure LVEF in this population these data were included in analysis”

**Comment 3**: Page 12, line 237: The part of the sentence that states: the success rate may have significantly improved….this should be deleted from results and only be in the discussion section.

**Response**:

Thank you for the recommended revision. We revised the manuscript as recommended. The sentence was deleted from the results and added to discussion.

“The device itself was less irritating to younger children then the process of finding the carotid pulse, and so it is possible the success rate may have significantly improved if the operator spent more time (e.g. 5 min) measuring for the waveform after giving the child time to settle”

**Comment 4:** On page 14 line 281 add: in children older than 5 years, the feasibility is not really proven for the younger set.

**Response:**

Thank you for the recommended revision. We have revised the manuscript to include the phrase “in children 5 years of age and older”

**Comment 5:** On page 15 lines 303-305: This is new information about the data being capturable in those less than 5 who held still or had their heads tilted. This should somehow be incorporated into the results when the data collection on this age group is reported. (I take it these were the 3-5 who had interpretable data)

**Response:**

Thank you for the recommended revision. We have revised the results section of the manuscript to include the sentence “Those patients were still and positioned with their heads tilted slightly to the side and upwards to expose the carotid artery better.”

**Comment 6:** On page 16 lines 314-315: Lack of differences between the pediatric cohorts for IF’s could also be due to numbers.

**Response:**

Thank you for the constructive comment. We agree with the reviewer’s comment that this is an important limitation. We have revised the discussion section of the manuscript to include the following statements in order to address the comments provided:

“A limitation of this study overall is the low number of patients enrolled and so future studies with larger patient enrollment will be important to verify the lack of difference seen between the pediatric cohorts in addition to the difference seen in specific pediatric cohorts in comparison to the adult population.”

**Comment 7**: On page 16, lines 321- 323: This study “suggests” not “demonstrates,” as it seems the differences were due to the youngest and oldest nonadult age group which seems odd.

**Response**:

We have revised the manuscript as requested.

**Additional response to editor:** Table 2 was not introduced in the text of the body. We added the following sentence to the results section to address this issue:

“Hemodynamics per pediatric age cohort are shown in Table 2.”
