# Supplementary material for: Proof-of-concept for a non-invasive, portable, and wireless device for cardiovascular monitoring in pediatric patients
Source: PLoS One. 2020 Jan 3;15(1):e0227145. doi: 10.1371/journal.pone.0227145 (PMC6941801; doi:10.1371/journal.pone.0227145)
Supplement: Attachment — Submitted filename: Response to Reviewers 12.9.2019.docx [file pone.0227145.s003.docx]

**AUTHORS’ RESPONSES TO REVIEWERS’ SUGGESTIONS**

**Manuscript #:** PONE-D-19-22751

**Title:** Proof-Of-Concept For A Non-invasive, Portable, and Wireless Device for Cardiovascular Monitoring in Pediatric Patients

**Authors:** Jennifer C. Miller, Jennifer Shepherd, Derek Rinderknecht, Andrew Cheng, Niema M. Pahlevan

We thank our editor and reviewers for the positive evaluations and constructive suggestions. Our manuscript has been greatly improved with the reviewers’ help. Below we list our responses in the sequence in which they were raised in each reviewer’s report.

**Comments to the Author**
Reviewer #1: The authors responded adequately to the reviewers' comments. The study provides important new insights.

**Response:**

We thank the author for their response

***Reviewer 2:***

**Comment 1:** The issues that I had previously brought up have been addressed within the limitations of the study. I have a couple of new comments.

Under study participants post transplant patients n=5 and post chemotherapy patients n=9. However in the results section post transpalnt n=6 and post chemo n=8 in the text and Table 1. Please reconcile.

**Response:**

We would like to thank the reviewer for this constructive comment. We have revised the section study participants to match the results section. Sup Fig 1 was also reconciled. The table and results discussion were reviewed and are correct.

**Comment 2**: Page 13 line 260: I think the authors mean B not A when referring to figure 3. "A" is used twice.

**Response:**

We thank the reviewer for their careful review. We revised the manuscript as recommended and changed the Fig 3 legend to list A and B.

**Comment 3:** The authors report that after manual review some additional waveforms could be added for assessment that the computer algorithm misclassified as not usuable. Were there any reversed by manual review, meaning the computer algorithm

**Response:**

We would like to thank the reviewer for this question. The automatic computer algorithm looks at the consistency of the recordings. For example, if there are 1-2 appropriate waveforms interspaced with poor recordings the algorithm will not capture them; however, a waveform expert can easily identify the good cycles in this situation. The reverse is highly unlikely. No computer identified recordings were later deemed by the expert reviewer to be inaccurate in this paper.
